# A Survey of Hesitancy and Response to the COVID-19 Vaccine Among Patients With Epilepsy in Northeast China

**DOI:** 10.3389/fneur.2021.778618

**Published:** 2021-11-17

**Authors:** Nan Li, Chaojia Chu, Weihong Lin

**Affiliations:** Department of Neurology, The First Hospital of Jilin University, Changchun, China

**Keywords:** COVID-19, vaccine hesitancy, epilepsy, side effect, China

## Abstract

**Objective:** This study was conducted to investigate the hesitancy and response of people with epilepsy (PWE) to the coronavirus disease 2019 (COVID-19) vaccine.

**Methods:** We conducted an online survey among PWE in northeast China about hesitancy and response to the COVID-19 vaccine. Their demographic background and symptomatic data about epilepsy were also recorded, and we analyzed the epilepsy-related risk factors in delaying the vaccine.

**Results:** In total, 357 patients with confirmed epilepsy were included in the survey, and only 38 (11%) patients received the COVID-19 vaccine. Fear of aggravating epilepsy (58%, *n* = 185), discouragement from health workers for epilepsy (22%, *n* = 70), and fear of patients of other unknown serious side effects (13%, *n* = 42) were the main reasons for delaying vaccination. A higher seizure frequency was the only epilepsy-related risk factor in delaying the vaccine (OR = 1.104, 95% CI: 0.988–1.233). None of the vaccinated patients reported that the vaccine aggravated their epilepsy.

**Significance:** Understanding concerns about the COVID-19 vaccine among PWE could help to improve health education and promote the establishment of an immune barrier.

## Introduction

In December 2019, coronavirus disease 2019 (COVID-19), caused by severe acute respiratory syndrome coronavirus 2 (SARS-CoV-2), broke out and led to a rapid worldwide pandemic. Up to the end of July 2021, COVID-19 had caused more than 192 million confirmed cases and over 4 million fatalities worldwide (1). To battle the virus, at least seven vaccines against COVID-19 are already available, and almost 300 candidate vaccines are in development (2). By July, more than 3,839 million COVID-19 vaccine doses had been administered globally (1). China, the first country to detect SARS-CoV-2, is also devoted to vigorously promoting the vaccination process, and more than 1,619 million doses have been given (3). In other words, more than half of the Chinese population has taken at least one dose.

Although the vaccine is thought to be safe for most people, patients with some preexisting conditions are still hesitant to accept the vaccine. Epilepsy is one of the most serious neurological disorders, with more than 70 million people worldwide suffering from this disorder (4). Approximately, 10 million patients are in China (5). Under the threat of COVID-19, people with epilepsy (PWE) are eager to know if they can be vaccinated safely and whether the vaccine can aggravate their disease. Therefore, we conducted an online survey among PWE in northeast China to investigate the hesitancy of PWE about the COVID-19 vaccine and whether the vaccine aggravated their condition.

## Methods

### Patient Recruitment

We conducted a cross-sectional survey in the Epilepsy Diagnosis and Treatment Center of the First Hospital of Jilin University on 1–19 July 2021 regarding the attitude and response of PWE toward the COVID-19 vaccine. The questionnaire was designed with WeChat and was released in the chat group consisting of patients with confirmed epilepsy who had previously visited the epilepsy outpatient clinic in our center. No advice from our physicians was given in the chat group before the survey. All patients in the chat group took part in the survey voluntarily. This implied that they consented when they completed the questionnaire. So, each enrolled patient provided informed consent.

The definitions of epilepsy and the classification of seizures conformed to the diagnostic criteria published by the International League Against Epilepsy (ILAE) (6, 7). Refractory epilepsy conformed to the criteria of 2010 (8). Active epilepsy was defined as having seizure occurrence within the preceding year, and inactive epilepsy was defined as reaching seizure-free status in the preceding year, regardless of whether antiseizure medications were administered. Focal to bilateral tonic-clonic seizures and general motor seizures were classified as convulsive seizures.

### Instruments

The questionnaire consisted of demographic background and symptomatic data on epilepsy (including the type of seizure, seizure frequency at present, and applied antiseizure medications). If the patients had received the COVID vaccine, they were asked about the possible common side effects (fever, fatigue, headache, nausea, and pain at the injection site) and whether the vaccine had aggravated their condition. If the patients had not been vaccinated, they were asked to describe their predominant concern about the vaccine.

As the vaccine applied in Jilin Province was the SARS-CoV-2 vaccine produced by the Beijing Institute of Biological Products Co., Ltd., (Sinopharm COVID-19 vaccine) or Sinovac Life Sciences Co., Ltd., (Sinovac COVID-19 vaccine), the responses to the vaccine after the first and second injections were interrogated separately.

### Statistical Analyses

Pearson's chi-squared test, the rank-sum test, and Fisher's exact test were used to compare continuous and categorical variables. Logistic regression was used to analyze the risk factors in delaying vaccination. Values for continuous variables are expressed as the means ± standard deviation (SD), and values for categorical variables are expressed as the frequencies (%). All *p* values were from two-tailed tests. *P* < 0.05 was considered to indicate statistical significance. The data were inputted and subsequently analyzed using SPSS for Windows, Version 24.0 (SPSS Inc., Chicago, IL, USA).

## Results

In total, 357 PWE answered to the survey and returned the questionnaire, and 38 (11%) patients received the COVID-19 vaccine. The flow chart demographic data are shown in Table 1. There were no significant differences between the vaccinated and unvaccinated groups in terms of age (Z = −0.313, *p* = 0.754) or sex (χ^2^ = 1.581, *p* = 0.209). The unvaccinated patients had a higher seizure frequency (Z = −1.979, *p* = 0.048). Fisher's exact test was executed, and no significant differences were found in the type of seizure (*p* > 0.05).

**Table 1:** Demographic information of people with epilepsy in the survey.

	**Vaccinated group (*n* = 38)**	**Unvaccinated group (*n* = 319)**	***p* value**
Age, y, M (IQR)^*^	33.5 (18)	33.0 (20)	0.754
Gender, N (%)			0.209
Male	17 (45)	176 (55)	
Female	21 (55)	143 (45)	
Current situation of seizure, N (%)			0.255
Active epilepsy	18 (47)	185 (58)	
Seizure frequency, N (%)			0.048
1–6 times per year	14 (78)	105 (57)	
6–12 times per year	1 (5.6)	23 (13)	
1–4 times per month	2 (11)	35 (19)	
4–20 times per month	0 (0.0)	16 (8.7)	
20–30 times per month	1 (5.6)	5 (2.7)	
Current type of seizure, N (%)			0.843
Focal aware motor seizure	2 (11)	20 (11)	
Focal aware nonmotor seizure	3 (17)	27 (15)	
Focal impaired awareness motor seizure	0 (0.0)	12 (6.5)	
Focal impaired awareness nonmotor seizure	3 (17)	32 (17)	
Focal to bilateral tonic-clonic seizure	10 (55)	87 (47)	
General motor seizure	0 (0.0)	6 (3.3)	
Inactive epilepsy	20 (53)	134 (42)	

* *M (IQR), median (interquartile range)*.

### Hesitancy About the COVID-19 Vaccine

The results of hesitancy are shown in Table 2. More than half of the unvaccinated patients delayed vaccination for fear of aggravating epilepsy, and this was the predominant concern. Discouragement from the health workers for epilepsy and fear of patients of other unknown serious side effects were the second and third reasons for delaying vaccination, respectively. Other diseases preventing them were uncontrolled hypertension (*n* = 3), malignant tumor (*n* = 2), systemic lupus erythematosus (*n* = 1), acquired immune deficiency syndrome (*n* = 1), congestive heart failure (*n* = 1), glucocorticoid applied for anti-NMDA-receptor encephalitis (*n* = 1), recent surgery (*n* = 1), hepatitis B (*n* = 1), and bedridden for cerebrovascular disease (*n* = 1). Among the unvaccinated patients, no significant differences were found in seizure frequency between the patients with fear of aggravating epilepsy or not (Z = −0.070, *p* = 0.944). Age, sex, seizure frequency, and convulsive seizure were set as independent variables in the logistic regression to analyze the epilepsy-related risk factors in delaying the vaccine, and a higher seizure frequency was the only risk factor (OR = 1.104, 95% CI:0.988–1.233).

**Table 2:** Predominant concerns about the COVID-19 vaccine among patients with epilepsy.

**Causes of hesitancy**	**Unvaccinated patients, N (%)**
Fear of aggravating epilepsy	185 (58)
Discouragement from health workers for epilepsy	70 (22)
Fear of other unknown serious side effects	42 (13)
Other diseases	12 (3.8)
Age limit	4 (1.3)
Breast-feeding	1 (0.3)
Recent human papillomavirus vaccination	1 (0.3)
Vaccine shortage	1 (0.3)
Others	3 (0.9)

### The Response to the COVID-19 Vaccine

**Figure 1:**
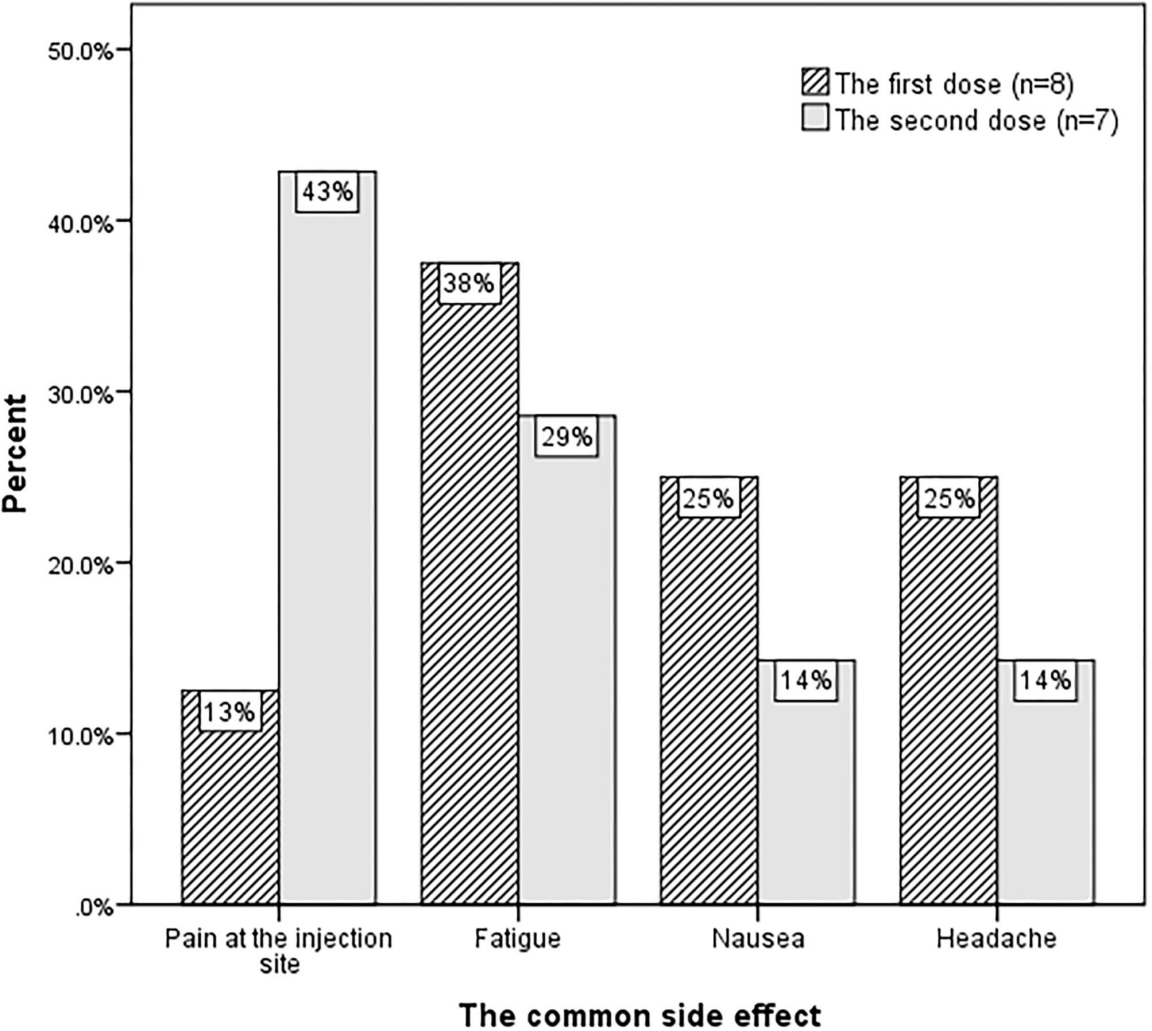
The common side effects of the COVID-19 vaccine in people with epilepsy.

Thirty-eight patients received the first dose of vaccine, and 22 patients completed the second dose. The antiseizure medications (ASMs) of vaccinated patients applied before the first vaccine are shown in Table 3. Five (13%) of the patients had refractory epilepsy. The majority had no side effects after the first dose (79%, *n* = 30) or the second dose (68%, *n* = 15). The common side effects are shown in Figure 1. No patients complained of fever. None of the vaccinated patients reported that the vaccine aggravated their epilepsy.

**Table 3:** Types of applied antiseizure medications of vaccinated patients, N (%).

**Applied antiseizure medication**	**Vaccinated patients (*n* = 38)**
Valproic acid	3 (7.9)
Oxcarbazepine	12 (32)
Topiramate	2 (5.3)
Levetiracetam	10 (26)
Phenobarbital	1 (2.6)
Lamotrigine	3 (7.9)
Lacosamide	1 (2.6)
Polytherapy	6 (16)

## Discussion

To our knowledge, this is the first study to investigate the hesitancy and response of PWE to the COVID-19 vaccine in China. PWE with a higher seizure frequency were prone to delay vaccination. Almost 90% of the PWE were hesitant to take the vaccine, and more than half of the PWE delayed the vaccination due to the fear of aggravating their epilepsy. Only 11% of the surveyed PWE accepted the vaccination, and none of them reported that the vaccine aggravated their condition of epilepsy.

As the COVID-19 vaccine is still being developed, several studies have been carried out to estimate its acceptance (9–11). A global survey reported that 71.5% of the participants were willing to take the vaccine, and China has the highest percentage (almost 90%) among the 19 countries (12). A nationwide study in China found that 83.3% of the people were definitely or probably willing to be vaccinated (13). Although the percentage varies among different social roles (14–17), most people have a positive attitude toward the vaccine. However, only 11% of the participants took the vaccine in our study, which was significantly lower than the national average uptake (3). This acceptance was also significantly lower than the data of Italian patients living with inflammatory bowel disease (80.3%) (18) and celiac disease (74.8%) (19). On one hand, the acceptance in our study referred to the rate of patients who had already received the vaccine, which might be lower than the rate of willingness. On the other hand, the pandemic was well-controlled in China when the survey was conducted, which may lead to lower enthusiasm. A higher seizure frequency was the only epilepsy-related risk factor in delaying the vaccine. Therefore, regular antiseizure medication treatment and timely drug adjustment may help to increase vaccine uptake among PWE.

Fear of aggravating epilepsy was the predominant reason for hesitancy about the vaccine. Without adequate experience of side effects of the novel vaccine, it is difficult for physicians to provide advice for patients. Consequently, patients would like to delay the vaccine until they receive a positive answer. In addition, it was recommended that the vaccine be used with caution among people with uncontrolled severe neurological disorders; thus, some health workers might discourage against vaccination for patients with active epilepsy. Although the sample size was limited, no side effects of aggravating epilepsy were reported, which is encouraging information for PWE. A national study with a large scale of PWE on the response to the COVID-19 vaccine is urgently needed.

As a novel vaccine, people are concerned about its safety and effectiveness, as are PWE. More than 10% of the patients worried about unknown severe side effects, which was also a negative driver in healthy people (9). Some mild self-limited side effects, which were common in normal people, also occurred in PWE. No patients complained of these mild side effects as a discouraging factor. Patients with serious diseases that may cause immune system dysfunction should delay vaccination (20). However, uncontrolled hypertension should not be a definite reason for rejecting the vaccine because many of the cases were due to unregulated blood pressure management rather than drug resistance. Along with the expansion of the applicable population, vaccines have recently become available to people more than 12 years old in many places in China, including Jilin Province. The patients who were previously prohibited by age limits can now gradually become vaccinated.

Our survey showed that most PWE would like to delay the COVID-19 vaccination, and the primary cause was the fear of aggravating their epilepsy. Timely drug adjustment and more positive attitude of physicians toward the vaccine may help to increase vaccine uptake among PWE.

## Limitation

To improve the enthusiasm of the patients to participate in the survey, the questionnaire was designed as concise as possible. But some accessional valuable information was not included in the questionnaire, such as anxiety or depression scale. No control group was included, and the patients with other chronic disease could be enrolled in the further study.

## Data Availability Statement

The raw data supporting the conclusions of this article will be made available by the authors, without undue reservation.

## Ethics Statement

The studies involving human participants were reviewed and approved by the Ethics Committee of the First Hospital of Jilin University. Written informed consent to participate in this study was provided by the participants' legal guardian/next of kin.

## Author Contributions

All authors listed have made a substantial, direct and intellectual contribution to the work, and approved it for publication.

## Conflict of Interest

The authors declare that the research was conducted in the absence of any commercial or financial relationships that could be construed as a potential conflict of interest.

## Publisher's Note

All claims expressed in this article are solely those of the authors and do not necessarily represent those of their affiliated organizations, or those of the publisher, the editors and the reviewers. Any product that may be evaluated in this article, or claim that may be made by its manufacturer, is not guaranteed or endorsed by the publisher.
